# *In vivo* targets of *Salmonella* FinO include a FinP-like small RNA controlling copy number of a cohabitating plasmid

**DOI:** 10.1093/nar/gkab281

**Published:** 2021-05-03

**Authors:** Youssef El Mouali, Milan Gerovac, Raminta Mineikaitė, Jörg Vogel

**Affiliations:** Institute for Molecular Infection Biology (IMIB), University of Würzburg, D-97080 Würzburg, Germany; Helmholtz Institute for RNA-based Infection Research (HIRI), Helmholtz Centre for Infection Research (HZI), D-97080 Würzburg, Germany; Institute for Molecular Infection Biology (IMIB), University of Würzburg, D-97080 Würzburg, Germany; Helmholtz Institute for RNA-based Infection Research (HIRI), Helmholtz Centre for Infection Research (HZI), D-97080 Würzburg, Germany; Institute for Molecular Infection Biology (IMIB), University of Würzburg, D-97080 Würzburg, Germany; Helmholtz Institute for RNA-based Infection Research (HIRI), Helmholtz Centre for Infection Research (HZI), D-97080 Würzburg, Germany

## Abstract

FinO-domain proteins represent an emerging family of RNA-binding proteins (RBPs) with diverse roles in bacterial post-transcriptional control and physiology. They exhibit an intriguing targeting spectrum, ranging from an assumed single RNA pair (FinP/*traJ*) for the plasmid-encoded FinO protein, to transcriptome-wide activity as documented for chromosomally encoded ProQ proteins. Thus, the shared FinO domain might bear an unusual plasticity enabling it to act either selectively or promiscuously on the same cellular RNA pool. One caveat to this model is that the full suite of *in vivo* targets of the assumedly highly selective FinO protein is unknown. Here, we have extensively profiled cellular transcripts associated with the virulence plasmid-encoded FinO in *Salmonella enterica*. While our analysis confirms the FinP sRNA of plasmid pSLT as the primary FinO target, we identify a second major ligand: the RepX sRNA of the unrelated antibiotic resistance plasmid pRSF1010. FinP and RepX are strikingly similar in length and structure, but not in primary sequence, and so may provide clues to understanding the high selectivity of FinO-RNA interactions. Moreover, we observe that the FinO RBP encoded on the *Salmonella* virulence plasmid controls the replication of a cohabitating antibiotic resistance plasmid, suggesting cross-regulation of plasmids on the RNA level.

## INTRODUCTION

Proteins that carry a FinO domain have recently garnered much attention as a new class of widespread RNA-binding proteins (RBPs) in bacteria (1,2). To date, several such proteins have been characterized, some of which in multiple species: FinO itself, FopA, ProQ and RocC. Genes of these proteins can be found in both, bacterial chromosomes (ProQ, RocC) and mobile genetic elements, primarily plasmids (FopA, FinO). The target suites of these RBPs, where determined, have turned out to be largely distinct from other well-characterized facilitators of post-transcriptional control, in particular, the RBPs CsrA/RsmA and Hfq (3–5).

*In vivo* interactome studies of ProQ, a prominent member of this class, found that this RBP recognizes hundreds of small noncoding RNAs (sRNAs) and mRNAs in *Escherichia coli, Neisseria meningitidis* and *Salmonella enterica* (6–9). *Legionella pneumophila* RocC was found to play a key role in the complex phenomenon of bacterial competence. Acting as an RNA chaperone, RocC uses its FinO domain to recognize an inhibitory sRNA (RocR) and several *trans*-encoded target mRNAs in the competence regulon (10,11). From these and other recent functional studies of ProQ (12,13) or ProQ-associated sRNAs (14,15), a working model has emerged whereby the FinO domain enables these RBPs to act in a transcriptome-wide manner to impact on various aspects of bacterial physiology. Whether the FinO domain recognizes a specific nucleotide sequence or structural element in these many cellular targets is currently unclear; no informative consensus binding motif could be extracted from the large target suite of *Salmonella* ProQ (7).

The global RBP activity of ProQ is in surprising contrast with the assumed narrow activity of the founding member of this family, the 22-kDa FinO protein encoded by IncF plasmids in *E. coli* (16,17). FinO was named so to reflect its fertility inhibition function observed during work on IncF plasmid conjugation (18). These IncF plasmids regulate their conjugation through an RNA antisense mechanism whereby the *cis*-encoded sRNA FinP inhibits protein synthesis of conjugation regulator TraJ (17,19,20). FinO serves a dual function in this regulation: it protects FinP from degradation by RNase E, and facilitates the inhibitory base pairing of FinP with *traJ* mRNA (21–23). Once formed, the FinP-*traJ* RNA duplex blocks access to the ribosome binding site (RBS) of *traJ* while the paired RNAs undergo co-degradation by RNase III (22). If FinO is absent, TraJ synthesis is derepressed, resulting in higher conjugation of the parental IncF plasmid (17,24,25).

While the regulation of IncF plasmid conjugation by FinO is well understood, it is less clear what determines the selectivity of FinO for its RNA targets. We know that the 79-nt FinP sRNA forms two stem loops, SLI and SLII, followed by a 3′ single-stranded uridine stretch (16,26). FinO seems to interact primarily with SLII and the 3′ tail (23), but SLI also contributes to overall binding affinity (27,28). Obviously, none of these RNA features is unique, and bacteria are rife with transcripts that possess two stem-loops and a 3′ uridine tail.

As efforts to understand the molecular basis of RNA target recognition and selectivity by FinO domain proteins gain new momentum (12,13,29,30), we must also determine the actual target suite of FinO itself. Based on early genetics (17,31), FinP and *traJ* have been regarded as the primary cellular ligands of FinO, yet this assumption has not been tested with global methods as used for FopA, ProQ or RocC (4,6,10). To address this open question, we have compared in *Salmonella enterica* serovar Typhimurium (henceforth, *Salmonella*) the cellular ligands of FinO and ProQ in a transcriptome-wide manner. On the one hand, we confirm that unlike the global RBP ProQ expressed from the *Salmonella* chromosome, the FinO protein of *Salmonella* plasmid pSLT has a restricted target suite. On the other hand, we make the surprise discovery of a yet another major sRNA target of FinO. Intriguingly, this sRNA to which we will refer as RepX originates from the replication control locus of an unrelated *Salmonella* plasmid. While distinct in terms of primary sequence, RepX is similar to FinP in length and RNA structure, which could help to understand the high specificity of FinO-RNA interactions. Most importantly, *Salmonella* FinO not only regulates the conjugation of its own plasmid, but through the RepX RNA also influences another mobile element in the same cell.

## MATERIALS AND METHODS

### Bacterial strains, plasmids and genetic manipulations

The bacterial strains and plasmids used in this study are listed in Supplementary Table S1. *Salmonella enterica* serovar Typhimurium SL1344 (32) and derivative strains were cultivated in Lysogeny broth (LB) (tryptone 10 g/l, yeast extract 5 g/l and sodium chloride 10 g/l). Bacterial cultures were inoculated to an OD_600 nm_ 0.001 and incubated at 37°C with 200 rpm shaking.

Deletion strains were generated by standard gene replacement as previously described (33). Similarly, epitope tagged proteins were constructed by following the standard protocol (34). When required, the antibiotic cassette was removed by the expression of Flp recombinase from pCP20 as previously described (35). Oligonucleotides used for strains construction are listed in Supplementary Table S1.

### Protein extracts and western blot

Bacterial cultures were grown to the desired conditions. A volume of cells that represents 1 OD_600 nm_ were pelleted and resuspended in 100 μl of 1× Laemmli buffer. Samples were stored at –20°C. Protein extracts were subjected to SDS-PAGE separation, transfer to PVDF filter and subsequent immunodetection. As primary antibodies, monoclonal anti-FLAG M2 (Sigma-Aldrich #F1804) 1:2000 for both FinO-3×FLAG and ProQ-3×FLAG detection, as loading control GroEL was detected with anti-GroEL (Sigma-Aldrich, #G6532), anti-mouse (Thermo Fisher, cat# 31430) and anti-rabbit (Thermo Fisher, cat# 31460) conjugated to horseradish peroxidase were used as secondary antibodies for anti-FLAG and anti-GroEL respectively. For detection, ECL™ Prime Western Blotting Detection Reagent (GE Healthcare) served as a substrate.

### Total RNA isolation and northern blot

Strains of interest were grown to the desired conditions. The biomass of 4 units of OD_600 nm_ was collected, and total RNA extracted by hot phenol method followed by a DNase treatment. Samples of 10 μg of total RNA were subjected to electrophoretic separation in Tris–borate–EDTA (TBE) 6% acrylamide gels containing 8.3 M urea. RNAs were transferred to Hybond N+ (GE Healthcare) filters. Transcripts of interest were detected by hybridization with 5′ radiolabeled oligos as probes. For oligonucleotide labeling, 10 pmol of the oligonucleotides used as probes were 5′-labeled with 10 μCi of ^32^P-γ-ATP by PNK (T4 polynucleotide kinase, Thermo Fisher Scientific) in a 20 μl reaction for 1 h at 37°C. Labeled oligonucleotides were ran over Microspin G-25 columns (GE Healthcare) to remove unincorporated ^32^P-γ-ATP. Radioactive signal was imaged with the Typhoon FLA 7000 (GE Healthcare). Oligonucleotides used as probes are listed in Supplementary Table S1.

### Rifampicin assay

Bacterial cultures were grown to OD_600 nm_ 2.0. At *t*_0_, a volume equivalent to 4 ODs of cells was collected for total RNA extraction. Rifampicin to a concentration of 500 μg/ml was added to the cultures to stop transcription. Samples at different time points were collected. A volume of 4 ODs of cells was added to a 14 ml falcon tube containing stop solution (95% ethanol 5% phenol) and snap frozen in liquid nitrogen. Samples were stored at –80°C and when processed, further subjected to total RNA extraction. Extracted RNA was visualized by northern blot. The stability of FinP and RepX was determined. Ribosomal 5S RNA was detected as loading control.

### Determination of relative plasmid copy number by quantitative PCR (qPCR)

The relative plasmid copy number of pRSF1010 was determined in *Salmonella enterica* WT and Δ*finO* as previously shown with minor modifications (36,37). Shortly, 2 ODs of cells were pelleted and resuspended in PBS 1× pH 7.4. Cells were lysed by boiling the samples at 95°C with immediate freezing. Samples were then diluted in PBS. The relative pRSF1010 copy number in WT and Δ*finO* was quantified by qPCR for 1 × 10^5^ cells by amplifying a 140 nt DNA site in the chromosome as reference (*crp* locus) and in pRSF1010 (*repA* locus) using Power SYBR Green (Applied Biosystems). Average quantification in WT samples was set to 1 to determine fold change in the Δ*finO* strain. Oligonucleotides used are listed in Supplementary Table S1.

### RNA-coimmunoprecipitation

RNA-coimmunoprecipitation was carried out as previously described (6,38). Briefly, strains carrying either FinO-3×FLAG or ProQ-3×FLAG were grown in LB to the desired OD_600 nm_. A volume of cells equal to 50 ODs was pelleted. Cells were resuspended in 800 μl of lysis buffer (20 mM Tris pH 8.0, 150 mM KCl, 1 mM MgCl_2_, 1 mM DTT) supplemented with 8 μl of DNase I. The suspension was then subjected to mechanical lysis by 0.1 mm glass beads at 30 Hz for 10 min in the Retsch MM200. The lysate was cleared by centrifugation. A volume equivalent to 0.5 OD_600 nm_ was diluted to 90 μl with 1× Laemmli buffer (lysate protein sample) and stored at −20°C. A volume equivalent to 5 OD_600 nm_ was saved for RNA extraction with TRIzol (lysate RNA sample). The remaining lysate was then incubated with 25 μl (1 μl/2 OD_600 nm_ of cells) of monoclonal anti-FLAG M2 (Sigma #F1804) at 4°C with rotation for 30 min. The sample was then added to 75 μl of pre-washed Protein A sepharose beads (Sigma-Aldrich) and further incubated with rotation at 4°C for an additional 30 min. The sample beads were spun down. From the supernatant, representing the flow through, a volume equivalent to 0.5 OD_600 nm_ was diluted to 90 μl with 1× Laemmli buffer (flowthrough protein sample) and stored at −20°C. A volume equivalent to 5 OD_600 nm_ was saved for RNA extraction with TRIzol (flowthrough RNA sample). Next, the beads were washed with lysis buffer for five times and protein and RNA was eluted from the beads. From the last wash, a volume equivalent to 0.5 OD_600 nm_ was diluted to 90 μl with 1× Laemmli buffer (wash protein sample) and stored at −20°C. A volume equivalent to 5 OD_600 nm_ was saved for RNA extraction with TRIzol (wash RNA sample). The beads were resuspended in 532 μl of lysis buffer. To a volume of 32 μl, 8 μl of 5× Laemmli buffer was added and stored at −20°C (elution protein sample). RNA-coimmunoprecipitated was extracted by phenol:chloroform:isoamyl alcohol (P:C:I) (25:24:1, pH 4.5, Roth) extraction. The purified RNA samples were treated by DNase I and purified by P:C:I extraction. Samples were resuspended in H_2_O to a final concentration of 1 OD/μl for elution samples and 0.1 OD/μl for lysate, flowthrough and wash. Resulting RNA samples were subjected to visualization via northern blot or quantification via deep sequencing. Protein extracts were further analyzed by western blot.

### cDNA library preparation

Library preparation from co-immunoprecipitated RNA samples were carried out with NEBNext Multiplex Small RNA Library Prep Set (New England Biolabs) that allow sequencing in Illumina platforms. The library preparation was carried out in a thermocycler and manufacturer's guidelines were followed with minor modifications. Briefly, 3.5 μl of RNA was mixed with 1 μl of 3′ SR Adaptor (1:10 diluted) and incubated at 70°C for 2 min. The tubes were transferred to ice and then, 6.5 μl of 3′ ligation mix (5 μl of 3′ ligation buffer, 1.5 μl of 3′ enzyme mix) were added to each tube and incubated at 25°C for 1 h (without lid heated). 2.75 μl of SR mix (2.5 μl of water, 0.25 μl of SR RT primer, 1:10 diluted) was added to the samples and incubated as follows: 75°C for 5 min (lid 85°C), 37°C for 15 min (no lid heated), 25°C for 15 min (no lid heated). For ligation of the 5′ adaptor the 5′ ligation mix was added to each sample (0.5 μl of 5′ ligation reaction buffer, 1.25 μl 5′ ligation enzyme mix and 0.5 μl of denatured 5′adaptor (1:5 diluted)) and incubated at 25°C for 1 hour (no lid heated).

To generate the first strand of cDNA 5 μl of the cDNA mix (4 μl first strand buffer, 0.5 μl Murine RNase inhibitor, 0.5 μl SuperScript II RT) was added to each reaction and incubated at 50°C for 1 h followed by 15 min at 70°C to inactivate the RT enzyme. 10 μl of cDNA was PCR amplified with barcoded NEB Index primer (1:10 diluted) and SR primer (1 :10 diluted) in a 50 μl volume reaction (25 μl LongAmp Taq 2× mix, 12.5 μl nuclease-free water, 1.25 μl SR primer, 1.25 μl index primer). The following PCR program was carried out: 30s at 94°C for initial denaturation, 15s at 94°C, 30s at 62°C and 70°C for 16 or 20 cycles, and a final elongation of 5 min at 70°C. PCR products were purified via AMPure XL beads. The distribution of the library and concentration of library DNA was determined by DNA bioanalyzer and a DNA Qubit measurement respectively. Amplified cDNAs from different libraries were pooled and sequenced on an Illumina NextSeq 500 platform at the Core Unit SysMed at the University of Würzburg.

### RIP-seq data analysis

Approximately 5 million single end 75 bp reads were sequenced per library. Generated FASTQ files were mapped to the *Salmonella enterica* serovar Typhimurium strain SL1344 genome: chromosome, NC_016810.1; three plasmids: pCol1B9, NC_017718.1; pRSF1010, NC_017719.1; pSLT, NC_017720.1. Annotation file of pRSF1010 plasmid was amended with the coordinates of the RepX sRNA prior to further data analysis.

Gene-wise read counts were normalized to TPM (transcripts per kilobase million) that takes into account sequencing depth (number of mapped reads) and transcripts length. Enrichment factors were calculated with DESeq2 (39). RNA coimmunoprecipitated with tagged FinO-3×FLAG or ProQ-3×FLAG (one biological duplicate each) was compared to the non-tagged WT strain to determine enrichment factors. DESeq2 utilizes Wald test to determine the *P*-value and the Benjamini-Hochberg to adjust *P*-values (*P*-adj) (39). Enrichment factors of log_2_fold 2.0 (4-fold) with *P*-adj <0.05 were considered significantly enriched for RIP-seq of ProQ-3×FLAG and FinO-3×FLAG at OD_600 nm_ 2.0 (Supplementary Tables S2 and S3). For samples of FinO-3×FLAG and ProQ-3×FLAG through the growth curve (OD_600 nm_ 0.15, 0.5, 2.0, 2.0 + 3 h, 2.0 + 6 h, 2.0 + 9 h) the read coverage normalized to TPM of transcripts enriched over 4-fold was used to represent FinO and ProQ occupancy through the growth curve (Supplementary Table S4).

### FinO purification

The purification of recombinant FinO was carried out using the expression plasmid pMiG007-His_6_-3C-FinO. Purification of FinO was carried out as previously described for FopA at the recombinant protein expression facility, Rudolf-Virchow-Center Würzburg (4).

### Preparation and labeling of RNA

FinP and RepX sRNA were *in vitro* transcribed in a T7 transcription reaction using the MEGAscript T7 Transcription kit (Thermo Fisher Scientific). As template, 200 ng of the mix of oligonucleotides JVO-18275/ JVO-18276 and JVO-18281/ JVO-18282 was used for FinP and RepX respectively. *In vitro* transcribed RNA was subjected to electrophoresis in Tris–borate–EDTA (TBE) 6% acrylamide gels containing 8.3 M urea. RNA bands of interest were excised from the gel and eluted in RNA elution buffer (0.1 M sodium acetate, 0.1% SDS, 10 mM EDTA) at 4°C overnight. Eluted RNA was further isolated via P:C:I extraction and precipitated in EtOH. Then, 50 pmol of RNA was dephosphorylated with 10 units of calf intestinal phosphatase (CIP, New England Biolabs) in a 50 μl reaction at 37°C for 1 h. Subsequently, CIP-treated RNA was again isolated by P:C:I extraction and EtOH precipitated. In a 20 μl reaction, 20 pmol of the dephosphorylated RNA was 5′-labeled with 20 μCi of ^32^P-γ-ATP by PNK (T4 polynucleotide kinase, Thermo Fisher Scientific) for 1 h at 37°C. Labeled RNA was further purified via Microspin G-50 columns (GE Healthcare) to remove unincorporated ^32^P-γ-ATP. Labeled RNA was subjected to an additional gel purification step, full length labeled FinP and RepX bands were excised from the gel and eluted in RNA elution buffer (0.1 M sodium acetate, 0.1% SDS, 10 mM EDTA) at 8°C overnight. Eluted RNA was further isolated via P:C:I extraction and precipitated in EtOH. Labeled RNA FinP* and RepX* was resuspended in H_2_O and stored at –20°C. Alternatively, *in vitro* transcribed RNA was labeled at the 3′ end as previously described (40). Shortly, the 3′ end of *in vitro* transcribed FinP and RepX were labeled with 3′,5′-cytidine (5′^32^P) diphosphate (pCp) and T4 RNA ligase I (New England Biolabs). To allow FinO binding, the 3′end of labeled FinP* and RepX* were dephosphorylated via an additional PNK dephosphorylating step as previously described (23). Dephosphorylated 3′-labeled FinP* and RepX* RNA was purified as described for the 5′-labeled species.

### Protein–RNA binding assays

For the electrophoretic mobility shift assay (EMSA), labeled RNA was denatured by heating at 95°C for 1 min and cooling the samples in ice. For binding, 4 nM of FinP* or RepX* were incubated in binding buffer (25 mM Tris–HCl pH 7.4, 150 mM NaCl, 1 mM MgCl_2_) with increasing concentration of FinO protein (0, 0.16, 0.32, 0.63, 1.25, 2.5, 5 μM) in the presence of 1 μg of Yeast RNA/reaction (Ambion) for 1 hour at 37°C. Reactions were stopped by adding 5× RNA native loading buffer (0.5x TBE, 50% glycerol, 0,2% xylene cyanol, 0.2% bromophenol blue) and separated on a native 6% polyacrylamide gels at 4°C in 0.5% TBE at constant current of 40 mA for 3–4 h. For visualization, gels were vacuum dried and signals detected on a Typhoon FLA 7000 phosphoimager (GE Healthcare). The apparent *K*_d_ of FinO binding to FinP or RepX was determined by quantifying the intensity of the bound and unbound fraction by FinO for FinP and RepX on ImageJ. The percentage of RNA bound was plotted as a function of FinO concentration and represented values were fitted to a curve followed by a nonlinear regression. The apparent *K*_d_ was determined as the concentration at which 50% of the labeled FinP* or RepX* is bound by FinO in our assay.

Where applicable, the binding of the labeled RNA (FinP* or RepX*) to FinO (500 nM) was competed with increasing concentration of non-radiolabeled counterpart RNA (FinP or RepX) (0.13, 0.25, 0.5, 1, 2 μM). The samples were incubated in binding buffer (25 mM Tris–HCl pH 7.4, 150 mM NaCl, 1 mM MgCl_2_) in the presence of 1 μg of yeast RNA/reaction (Ambion) for 1 h at 37°C. Reactions were stopped by adding 1× RNA native loading buffer and separated and visualized as described above. Alternatively, the binding of FinP* to FinO was further assessed by classic filter binding assay (41). Radiolabeled FinP* (4 nM) was incubated with increasing concentration of FinO (0, 0.63, 1.25, 2.5, 5 μM) in binding buffer (25 mM Tris–HCl pH 7.4, 150 mM NaCl, 1 mM MgCl_2_) in the presence of 1 μg of Yeast RNA/reaction (Ambion) for 30 min at 37°C. The binding of the labeled FinP* to FinO (5 μM) was competed with increasing concentration of unlabeled counterpart RNA, i.e. RepX or RepX C-U (0.13, 0.25, 0.5, 1, 2 μM). Samples were filtered through a 0.2 μm pore size nitrocellulose membrane (Amersham™, GE Healthcare) and a Hybond+ membrane in a Dot blot apparatus. For washing, 200 μl of binding buffer in absence of yeast RNA was used. FinO bound to RNA is visualized as immobilized RNA in the nitrocellulose membrane while unbound RNA is visualized as bound RNA to the Hybond+ membrane.

### RNA structure probing

RNA structure probing of FinP* and RepX* was carried out as previously described (4,14). Shortly, labeled RNAs were prepared as described for the EMSA. For the reactions, 0.4 pmol of labeled RNA were denatured and incubated with FinO at 5 μM concentration when indicated for 15 min at 37°C in the presence of 1× binding buffer (25 mM Tris–HCl pH 7.4, 150 mM NaCl, 1 mM MgCl_2_) and 1 μg of yeast RNA in 10 μl. The reactions were then left untreated (Ctr) or treated with 5 mM lead acetate (PbII) (14). RNA was then P:C:I extracted and EtOH precipitated. RNA was resuspended in H_2_O. For the OH ladder, 1 pmol of labeled RNA was denatured at 95°C for 5 min in 1× alkaline buffer (Ambion) in a 10 μl reaction. For the T1 ladder, 1 pmol of RNA was denatured in water for 1 min at 95°C followed by addition of RNase T1 enzyme and incubated for 3 min at 37°C. All reactions were stopped by addition of GLII RNA loading buffer (95% deionized formamide, 0.02% SDS, 0.02% bromphenol blue, 0.01% xylene cyanol and 1 mM EDTA). Samples were boiled at 95°C and RNA was phenol:chloroform:isoamyl alcohol (P:C:I) (25:24:1, pH 4.5, Roth) extracted and ethanol precipitated. Samples were resuspended in H_2_O and mixed with GLII RNA loading buffer. Samples were then boiled at 95°C and loaded on a 10% polyacrylamide 7 M urea gel and separated for 3 h at 45 W. As for the EMSA, for visualization, gels were vacuum dried and signals detected on a Typhoon FLA 7000 phosphoimager (GE Healthcare).

### FinP and RepX consensus sequences and structure prediction

FinP and RepX sequence from pSLT and pRSF1010 respectively was used as input in GLASSgo (42,43). Output sequences were manually filtered and aligned with LocARNA (43,44). The resulting alignment in Stockholm format was used as input in R-scape to determine and evaluate consensus structures for FinP and RepX that are visualized by R2R (45,46). Alignment of FinP and RepX sequences was visualized in Jalview (47). FinP, RepX and ProQ dependent sRNAs structures in Supplementary Figure S4 were predicted by minimum free energy (MFE) and partition function of RNAfold (48) and visualized in VARNA (49).

## RESULTS

### Global mapping of FinO targets *in vivo*

**Figure 1.**
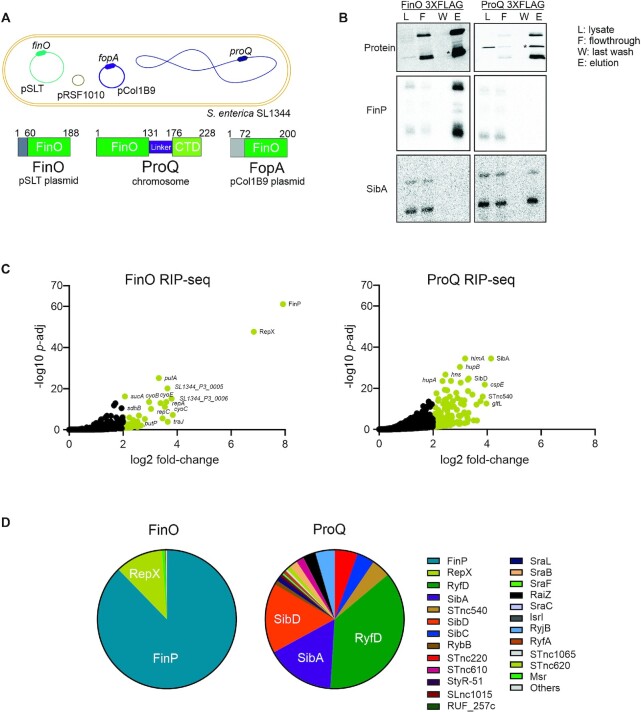
Global mapping of FinO targets *Salmonella enterica* SL1344. (**A**) Schematic representation of genetic elements present in *S.enterica* SL1344, the chromosome and the three plasmids pSLT, pCol1B9 and pRSF1010. Below, schematic representation of pSLT plasmid encoded protein FinO, chromosome encoded protein ProQ and pCol1B9 plasmid encoded FopA. (**B**) Upper panel, detection by western blot of immunoprecipitated FinO-3×FLAG and ProQ-3×FLAG. Lower panels, direct detection by northern blot of co-enriched FinP and SibA sRNAs in ProQ and FinO RNA co-immunoprecipitation samples. (**C**) Volcano plot of RNA transcripts enriched by RIP-seq of FinO-3×FLAG (left panel) and ProQ-3×FLAG (right panel). RNA coimmunoprecipitated with tagged FinO-3×FLAG or ProQ-3×FLAG was compared to the non-tagged WT strain to determine enrichment factors. (**D**) Pie chart representing the read distribution of sRNAs enriched by FinO and ProQ on RIP-seq samples from panel C.

To study FinO targets *in vivo*, we chose the *Salmonella enterica* model strain SL1344, which carries three plasmids: pSLT, pCol1B9 and pRSF1010 (Figure 1A). The homolog of *E. coli* FinO is located on the so-called virulence plasmid pSLT, which carries the virulence-associated *spv* operon (50). This 95 kb plasmid encodes its own conjugation machinery and therefore is self-transmissible (51). By way of inference from *E. coli*, FinO of *Salmonella* represses the conjugative transfer of pSLT by interacting with the pSLT-encoded *traJ* mRNA and its antisense RNA, FinP, protecting FinP from degradation and facilitating sense-antisense RNA recognition. *Salmonella* FinO is a 188-aa (∼21-kDa) protein, thus slightly smaller than the ProQ protein (228 aa, ∼25 kDa), which is expressed from the *Salmonella* chromosome (Figure 1A).

To obtain a comprehensive map of FinO ligands *in vivo*, we followed the procedure established for global analysis of ProQ ligands (6), which uses RNA co-immunoprecipitation (coIP) followed by RNA-seq (RIP-seq). To this end, we tagged the *finO* open reading frame on the pSLT plasmid with a C-terminal FLAG epitope and confirmed successful tagging by western blot analysis of the resultant *finO*::3×FLAG strain (Supplementary Figure S1A). Testing functionality of the tagged FinO protein in the coIP step, we performed northern blot analysis to detect the FinP (75 nt) or SibA (138 nt) sRNAs, the latter of which is an abundant cellular ligand of ProQ (6-8). As shown in Figure 1B, FinO coIP enriched FinP but not SibA, whereas ProQ coIP with a *proQ*::3×FLAG strain showed the expected reciprocal enrichment pattern.

Next, we performed RIP-seq in the early stationary phase of growth (OD_600 nm_ of 2.0), i.e., our standard condition in several previous RBP studies (6,7,52,53) (Supplementary Figure S1B-C). A *proQ*::3×FLAG strain was analyzed in parallel, to permit a direct comparison of these two RBPs. Deep sequencing of the coIP RNA report a strong (∼250-fold) enrichment of FinP with FinO (Figure 1C). Indeed, FinP contributed 82% of all reads (cleaned for rRNA) from the FinO coIP library. As expected, the *traJ* mRNA of pSLT was also enriched (∼14-fold), but was covered by few reads (0.007%), which is likely due to its overall low cellular abundance.

Unexpectedly, the FinO coIP reported very strong (∼130-fold) enrichment of another transcript: the 74-nt RepX sRNA expressed from pRSF1010, i.e., the third plasmid in strain SL1344 (Figure 1C). RepX contributed >10% of all reads in the FinO coIP library. Considering only sRNA reads, FinP (87%) and RepX (12%) accounted for 99% of these cDNAs (Figure 1D).

Contrasting the enrichment of these two plasmid sRNAs by FinO, the ProQ coIP showed the expected enrichment of chromosomally encoded sRNAs, with SibA, SibD and RyfD being most abundant (6) (Figure 1D). We note that RyfD is more and SibC less prominent than in our previous study (6). This may be due to slight changes in the growth conditions and media, as well as the normalization procedure (6). Overall, however, these initial RIP-seq results again proved ProQ expressed from the chromosome as a global RBP, whereas the related plasmid-encoded FinO protein acting in the same cytoplasm has a more restricted target suite. However, the FinO targetome was not restricted to FinP-*traJ* and included a previously unknown major ligand, the RepX sRNA.

### FinP and RepX are the major ligands of FinO independent of growth stage

**Figure 2.**
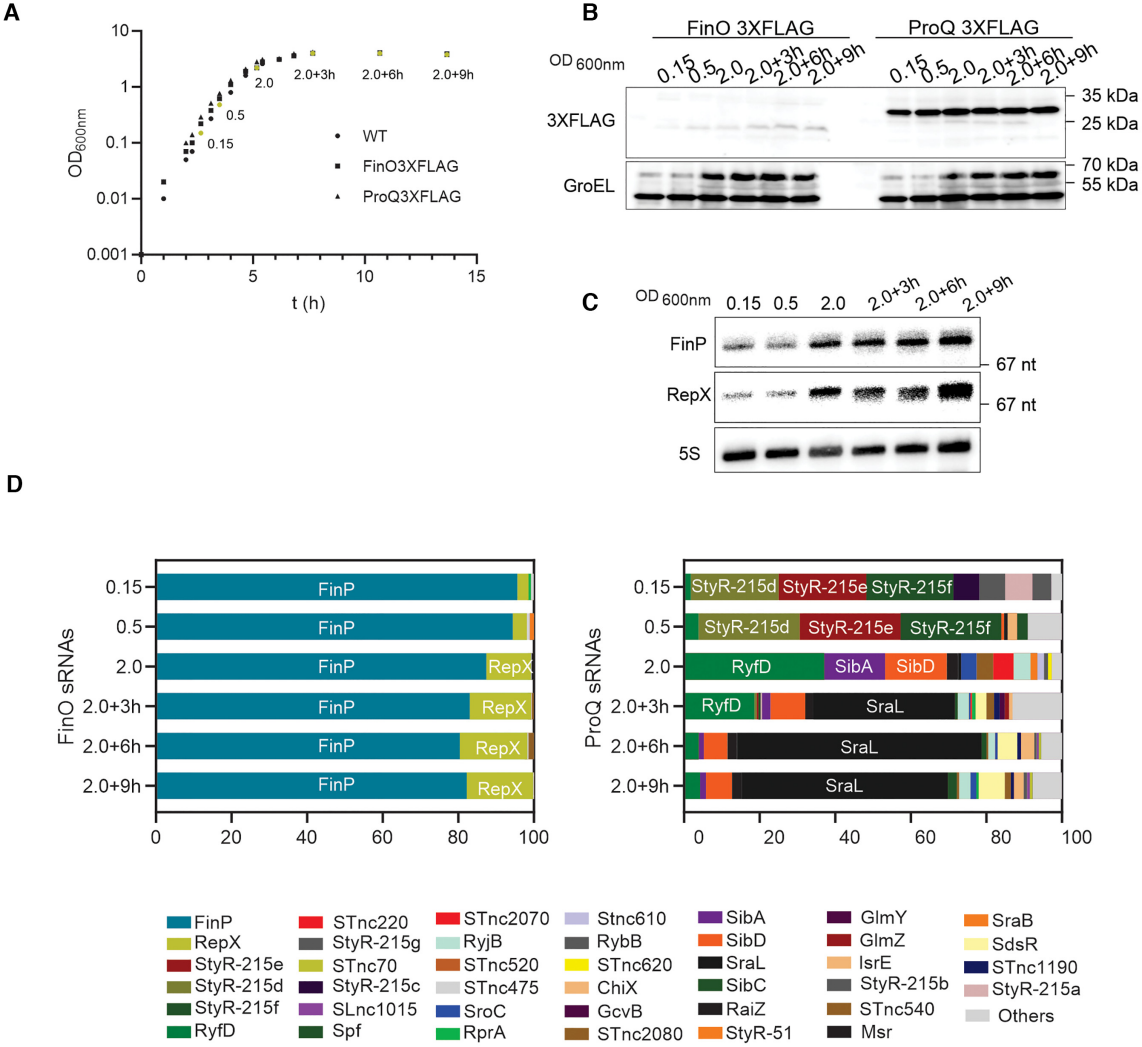
Growth phase dependent sRNA enrichment of FinO and ProQ. (**A**) Growth curve *Salmonella enterica* SL1344 WT and strains carrying FinO-3×FLAG or ProQ-3×FLAG. In yellow indicated time points selected for downstream experiments (OD_600 nm_ 0.15, 0.5, 2.0, 2.0 + 3 h, 2.0 + 6 h, 2.0 + 9 h). (**B**) FinO and ProQ expression level determined by western blot detection of 3×FLAG variants at OD_600 nm_ 0.15, 0.5, 2.0, 2.0+3h, 2.0+6h, 2.0+9h. GroEL was detected as loading control. (**C**) Northern blot detection in WT strain of RepX and FinP expression level through the growth curve (OD_600 nm_ 0.15, 0.5, 2.0, 2.0 + 3 h, 2.0 + 6 h, 2.0 + 9 h). Ribosomal 5S RNA was detected as loading control. (**D**) RIP-seq of FinO-3×FLAG and ProQ-3×FLAG through the growth curve (OD_600 nm_ 0.15, 0.5, 2.0, 2.0 + 3 h, 2.0 + 6 h, 2.0 + 9 h). Read distribution of sRNAs co-enriched by FinO (left panel) and ProQ (right panel).

The above RIP-seq analysis provided a snapshot of the FinO ligands in a single phase of growth. To analyze FinO ligands more comprehensively, we performed RIP-seq at five additional points along the bacterial growth curve, from early exponential growth into deep stationary phase (Figure 2A). Using western blot detection, we observed ProQ being generally more abundant than FinO in all conditions throughout the experiment. FinO accumulates at later time points while no major changes are observed for ProQ (Figure 2B). FinP and RepX were detected by northern blot analysis of total RNA samples taken along the growth curve. Either sRNA increases in abundance as growth rate decreases, with RepX showing the stronger increase of the two (Figure 2C).

The coIP time-course showed that FinP and RepX are indeed the major FinO ligands in all tested growth phases (Figure 2D). Of note, RepX seems to be more abundant upon entry into stationary phase. The fraction of RepX associated with FinO increases from <5% at the two early growth phases to over 15% as *Salmonella* enters late stationary phase. FinP is the most abundant target in all conditions, with 95% of the reads at the two early growth phases to over 80% at later time points. Altogether, FinP and RepX represent over 99% of the enriched sRNAs by FinO in all growth conditions.

ProQ coIP revealed a much more dynamic picture than the largely growth-phase independent target profiles of FinO. Different from the snapshot taken at early stationary phase (Figure 1D), the cDNA libraries from the two early growth phases are dominated by three distinct sRNAs (StyR-d, StyR-e, StyR-f). Upon entry into stationary phase, the occupancy of ProQ shifts towards the sRNA SraL, which then accounts for ∼50% or more of the ProQ ligands (Figure 2D). The here-observed strong association of this RpoS-dependent stationary-phase specific sRNA (54,55) lends support to a previously proposed activity of SraL as a ProQ dependent regulator of *rho* mRNA (56). Interestingly, although ProQ is more abundant than FinO (Figure 2B), in none of the growth conditions tested did ProQ enrich FinP or RepX, indicating that these sRNAs constitute unique high affinity targets of FinO.

### RepX as new ligand of FinO

**Figure 3.**
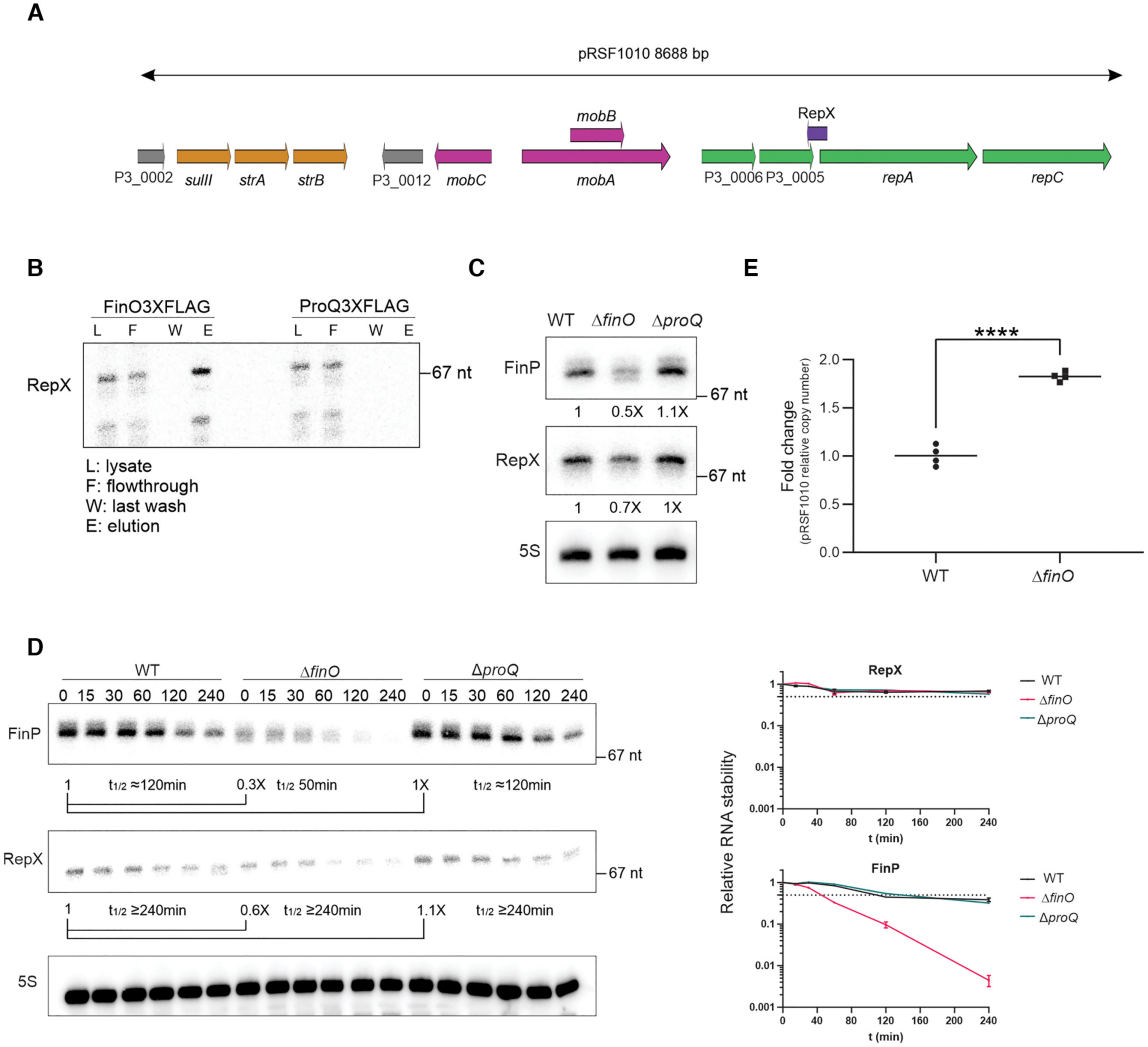
RepX as new ligand of FinO. (**A**) Schematic representation of pRSF1010 plasmid present in SL1344. In yellow antibiotic resistance cassetes to streptomycin and sulfonamides. In pink, loci involved in mobilization of the plasmid. In green loci involved in replication of the plasmid. In grey loci with unknown function. RepX in purple antisense to *repA* locus. (**B**) Northern blot detection of RepX co-immunoprecipitation with FinO or ProQ. (**C**) Total RNA levels of FinP and RepX levels in WT, Δ*finO* and Δ*proQ* strains detected by northern blot. 5S was detected as a loading control. (**D**) Stability of FinP and RepX in WT, Δ*finO* and Δ*proQ* strains determined by detection of FinP and RepX by northern blot after rifampicin treatment. Samples were collected at time points 0, 15, 30, 60, 120 and 240 min. Right panels, quantifications of FinP and RepX stability. (**E**) Relative plasmid copy number of pRSF1010 in WT and Δ*finO* strains. The ratio plasmid pRSF1010/chromosome was quantified by qPCR in WT and Δ*finO* extracts from cultures at OD_600 nm_ 2.0. Average of WT values was set to 1 to determine fold change in Δ*finO* extracts. Unpaired two-tailed Student's *t*-test was carried out, ****P < 0.0001.

RepX is an antisense sRNA that overlaps with the RBS and initiation codon of the *repA-repC* mRNA encoding replication proteins of the pRSF1010 plasmid (Figure 3A) (57–60). The RIP-seq results suggested that RepX, just like FinP, was selectively enriched by FinO and not by ProQ. To validate these deep sequencing-based results with an independent method, we performed northern blot analysis of the FinO and ProQ coIP samples (Figure 3B), and observed the expected enrichment of RepX by FinO and absence of RepX enrichment by ProQ in the eluate fraction.

Based on work in *E. coli*, FinO protects the FinP sRNA from RNase E-mediated degradation and therefore positively regulates its stability (22). Accordingly, we observed the steady-state level of FinP to be lower in a *Salmonella* Δ*finO* strain, as compared to WT. By contrast, a Δ*proQ* mutation did not affect FinP levels (Figure 3C). Importantly, the same pattern was observed with RepX; reduced levels in the absence of FinO but not of ProQ (Figure 3C). Whether or not FinO stabilizes RepX turned out to be difficult determining since rifampicin treatment assays showed the RepX sRNA to be exceptionally stable over the course of 240 min, regardless of whether WT, Δ*finO* and Δ*proQ* bacteria were probed (Figure 3D). The same samples probed for the FinP sRNA, however, did show a FinO-dependent stabilization (Figure 3D), arguing that FinO’s protective role is conserved in *Salmonella* (25).

As an alternate approach to addressing whether FinO is required for RepX functions, we looked into a RepX-mediated process, i.e., copy number control of the pRSF1010-like plasmid (57,58). Specifically, we determined the relative copy number of pRSF1010 by quantitative PCR (qPCR) in WT and Δ*finO* cells in early stationary phase (OD_600 nm_ of 2.0), using the ratio between pRSF1010 plasmid DNA and chromosomal DNA as a proxy for specific plasmid abundance in the cell. We observed an increased plasmid/chromosome ratio in the Δ*finO* strain as compared to WT, suggesting that absence of FinO alleviates the RepX-mediated repression of pRSF1010 replication (Figure 3E). Thus, through its positive effect on RepX, FinO regulates the synthesis of a mobile genetic element unrelated to the originally described conjugation control of its own plasmid, here pSLT.

### FinO shows specificity for FinP-like structured sRNAs

**Figure 4.**
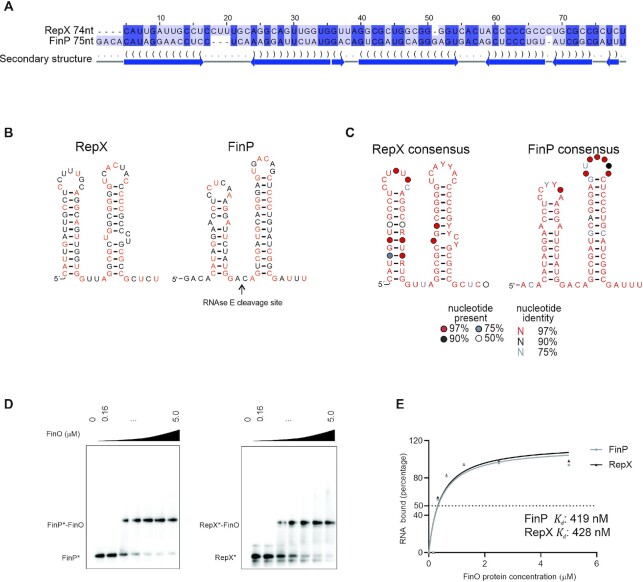
FinO specificity for FinP-like structured sRNAs. (**A**) FinP and RepX sequences from pSLT and pRSF1010 were aligned in LocARNA (44) and visualized on Jalview (47). (**B**) Predicted structure of FinP and RepX sRNAs. In orange indicated nucleotides that are conserved between FinP and RepX. (**C**) RepX and FinP consensus structure from alignment in Supplementary Figure S2. (**D**) EMSA with FinO of FinP and RepX sRNAs. Radiolabeled FinP (left panel) or RepX (right panel) (4 nM) was incubated with increasing concentration of FinO (0, 0.16, 0.32, 0.63, 1.25, 2.5, 5 μM). (**E**) Quantification of FinO binding affinity to FinP and RepX. The apparent *K*_d_ of FinO binding to FinP or RepX was determined by quantifying the intensity of the bound and unbound fraction by FinO for FinP and RepX on ImageJ. The percentage of RNA bound is plotted as a function of FinO concentration. The values were fitted to a curve followed by a nonlinear regression. The apparent *K*_d_ was determined as the concentration at which 50% of the labeled FinP* or RepX* is bound by FinO in our assay.

Since the coIP experiments showed FinP and RepX to be the preferred cellular ligands of FinO, we hypothesized that these two sRNAs possess similar features. Indeed, *in silico* RNA alignment and structure prediction suggested that they adopt a similar fold of two stem-loops (Figure 4A). A closer inspection revealed that even on the level of primary sequence, FinP and RepX show nearly 50% identity (Figure 4A). These shared nucleotides are spread over the entire sRNA sequences rather than constituting a conserved RNA stretch. Importantly, the predicted RepX RNA structure also shows a 3′ single stranded tail (Figure 4B), a feature shown to be important for recognition of FinP by FinO (23). There are notable differences, too. RepX lacks the short 5′ extension of FinP as well as the RNase E cleavage site that is located in the linker region between the two stem–loops of FinP (Figure 4B). Specifically, the GACA spacer (FinP), which is recognized and cleaved by RNase E (22), reads GUUA in RepX (Figure 4B). We speculate that this difference in sequence might be responsible for the much higher stability of RepX as compared to FinP, and the mild effect of FinO on RepX decay (Figure 3D).

To gauge the importance of the above sequence features of FinP and RepX, we sought to understand to which extent they are variable in potential homologs of these sRNAs. While the identification of plasmid sequences in genome repositories is not trivial, the use of GLASSgo (42) allowed us to predict 24 RepX-like sequences, five of which were unique (Supplementary Figure S2). The same approach applied to FinP retrieved over five hundred sequences, eleven of the 24 top sequences being unique (Supplementary Figure S2). Sequence alignments and structure predictions based on these two data sets argue that the overall two-stem-loop structure is a major conserved feature of the FinP and RepX sRNAs (Figure 4C).

The *cis-*antisense targets of the two sRNAs were also invariable. *Salmonella* RepX is located antisense to *repA* on the pRSF1010 plasmid (Figure 3A), and the *repA* mRNA, too, is bound by FinO (∼14-fold enrichment in coIP) (Figure 1C). The RepX antisense region within the *repA* mRNA can be folded into two stem loops that mirror SLI and SLII of RepX (Supplementary Figure S3B). Thus, in both the FinP-*traJ* and RepX-*repA* pairs the sRNA sequesters the translation initiation region of the target gene by a similar antisense mechanism (Supplementary Figure S3A-B), indicating mechanistic similarity in the FinP-mediated control of pSLT conjugation and RepX-mediated control of pRSF1010 replication.

### Hierarchy of FinO ligands recapitulated *in vitro*

Despite the structural similarity of FinP and RepX, FinP is the primary ligand of FinO *in vivo* (Figure 1D). This could be due to a higher synthesis rate of FinP (note that stability is similar; see Figure 3D) or a higher affinity of FinO to FinP than to RepX. To determine affinities, we performed gel mobility shift assays with FinO and *in vitro* synthesized, 5′ labeled FinP and RepX. Interestingly, FinO displayed similar apparent affinity (∼500 nM) for FinP and RepX in this type of binding assay (Figure 4D-E).

**Figure 5.**
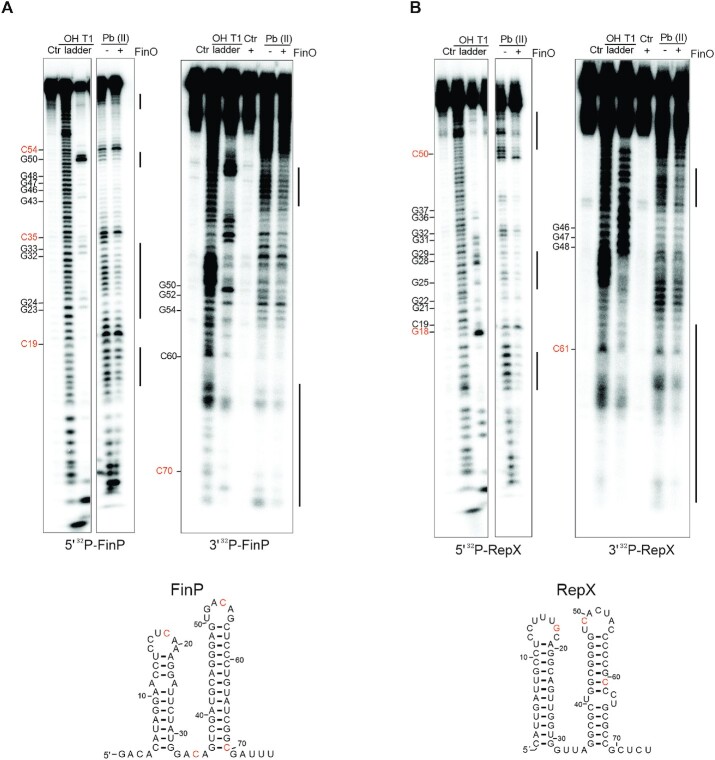
Mapping of FinO interactions with FinP and RepX. (**A**) Structure probing of FinP, either 5′ (left) and 3′ (right) labeled, in presence of FinO. FinP* labeled samples was left untreated for the control lane (Ctrl). FinP* was denatured and treated by RNase T1 or alkaline hydrolysis to generate T1 and OH ladders respectively. FinP* in the absence (−) or presence (+) of FinO (5 μM) was treated with lead acetate (Pb(II)). (**B**) Structure probing of RepX, either 5′ (left) and 3′ (right) labeled, in presence of FinO. RepX* labeled sample was left untreated for the control lane (Ctr). RepX* was denatured and treated by RNase T1 or alkaline hydrolysis to generate T1 and OH ladders respectively. RepX* in the absence (−) or presence (+) of FinO (5 μM) was treated with lead acetate (Pb(II)). For orientation, the relative positions of nucleotides in the ladders are highlighted in orange in the secondary structures of FinP and RepX.

We used *in vitro* structure probing to determine potential differences in FinO binding to these two sRNAs. In previous analysis of *E. coli* FinO with FinP (23), only the SLII of FinP and not the full length transcript was used as target RNA. In that setup, FinO interacted primarily with the base of the stem loop and the 3′ tail of SLII of FinP (23). While FinO interacts with the SLII of FinP with higher affinity than with SLI, it binds the full length FinP with even higher affinity (27). For this reason, we here decided to probe the full-length *Salmonella* FinP and RepX sRNAs (Figure 5A, B).

Probing with lead acetate (Pb(II)), which cleaves single stranded RNA, confirms that FinP forms two stem loops (Figure 5A). Prominent cleavage by Pb(II) is observed near positions C_19_, C_35_ and C_54_ indicated in orange, all bases located at single stranded regions in the predicted structure of FinP (Figure 5A). No digestion is observed in the stem region of SLII of FinP, while partial digestion is observed in the stem region of SLI, thus indicating that SLII structure is more stable than of SLI (Figure 5A). In the presence of FinO, the putative double stranded regions of SLI and SLII are further protected from Pb(II) cleavage, particularly noticeable for SLI (Figure 5A). RepX digestion profile by Pb(II) follows a similar trend to FinP, indicating a two-stem–loop structure with single stranded loop region near C_18_ and C_50_ (Figure 5B). In contrast to FinP, similar Pb(II) cleavage is observed for both SLI and SLII. In the presence of FinO, a reduced digestion profile is observed in the stem region of both SLI and SLII of RepX (Figure 5B).

As mentioned above, FinO has been shown to contact the 3′ tail of SLII in FinP (23). To visualize how FinO interacts with the 3′ end of FinP and RepX, *in vitro* structure probing was also carried out with 3′ end-labeled versions of these RNAs. As shown in Figure 5A, the 3′ tail of FinP, downstream of C_70_, is mildly protected from Pb(II)-induced cleavage in the presence of FinO. A protection of the base of SLII and the 3′tail, downstream of C_61_, is also observed for RepX (Figure 5B).

The observed FinO-dependent suppression of lead acetate-induced cleavage might be a direct consequence of protein binding, i.e., by FinO directly shielding the sRNA regions in question from the RNA cleavage agent or be a secondary effect by FinO rendering the sRNAs structure more stable. In addition, the described interaction of FinO with the 3′ end of FinP seems to occur similarly in RepX. Overall, FinO seems to interact with FinP and RepX in a similar manner, stabilizing their secondary structures by interaction with the stems of SLI and SLII and the 3′ end tail (Figure 5A, B).

**Figure 6.**
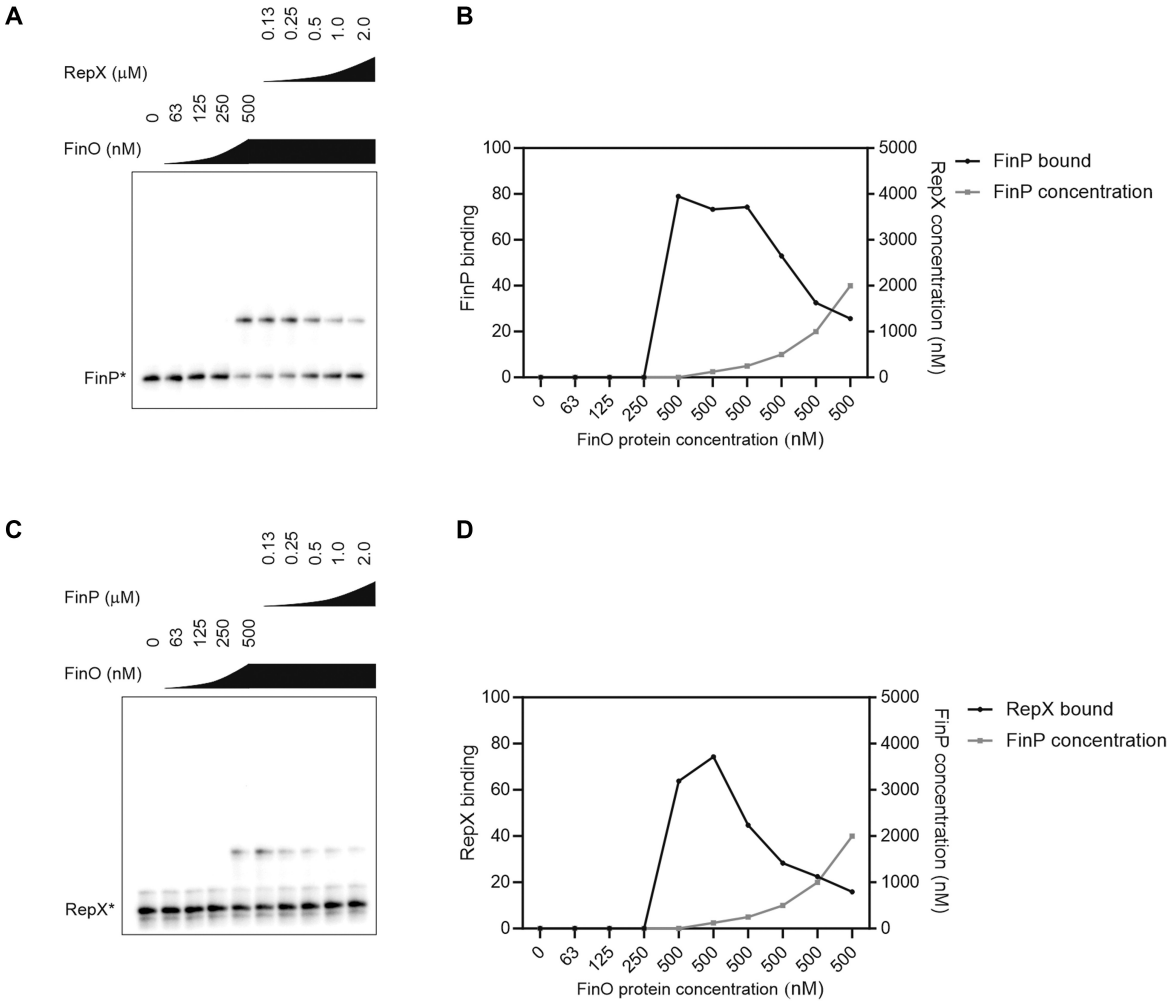
FinP and RepX competition for FinO binding. (**A**) EMSA with FinP competing RepX from FinO binding. Radiolabeled RepX (4 nM) was incubated with increasing concentration of FinO (0, 63, 125, 250, 500 nM). In the presence of 500 nM of FinO, the binding was competed with increasing concentration of non-radiolabeled FinP (0.13, 0.25, 0.5, 1, 2 μM). (**B**) Quantification of panel A. (**C**) EMSA with RepX competing FinP from FinO binding. Radiolabeled FinP (4 nM) was incubated with increasing concentration of FinO (0, 63, 125, 250, 500 nM). In the presence of 500 nM of FinO, the binding was competed with increasing concentration of non-radiolabeled RepX (0.13, 0.25, 0.5, 1, 2 μM). (**D**) Quantification of panel C.

Finally, we did observe preference of FinO for FinP as a ligand when competing the two sRNAs in a gel mobility shift assay. To this end, radiolabeled FinP or RepX sRNAs were subjected to binding by FinO, upon which unlabeled RepX or FinP, respectively were added (Figure 6). First, as control, labeled FinP* and RepX* were competed with the unlabeled RNA FinP or RepX RNAs, respectively (Supplementary Figure S4A-B). Both FinP and RepX outcompeted its labeled counterpart in a concentration-dependent manner (Supplementary Figure S4A, B). When competing FinP and RepX, we observed that addition of RepX displaced FinP* from its complex with FinO, however FinP outcompeted RepX* more effectively as the displacement of RepX from FinO by FinP occurred at a lower FinP concentration (Figure 6). When the competition was carried out with an increased FinO concentration (5 μM), FinP outcompeted RepX from FinO while RepX was unable to compete with FinP for binding to FinO (Supplementary Figure S4A, B). Taken together, these experiments suggest that FinP might be the preferred ligand of FinO.

## DISCUSSION

The study of FinO domain proteins presents unique opportunities to understand how RBPs achieve specificity in post-transcriptional control circuits by their ability to select a distinct set of sRNA and mRNA targets from thousands of (often more abundant) other transcripts in the cell. FinO, the founding member of this RBP class, was particularly interesting for its previously assumed specialized activity on two transcripts, the *traJ* mRNA and its antisense regulator FinP sRNA. How can its FinO domain be so selective when the same domain renders ProQ a truly global RBP with preference for transcripts from the chromosome? In the long-term quest to answer this central question, it was crucial to determine the target suite of FinO *in vivo*. While we can confirm the FinP sRNA as the major RNA interactor of FinO, we also discover a FinO-bound structural FinP-lookalike sRNA from a fully unrelated regulatory circuit on a different plasmid, which would have been nearly impossible without applying a global method such as RIP-seq.

As discussed below, our discovery of RepX provides a starting point for high-throughput characterization of the essential RNA features for recognition by FinO. Moreover, the *in vivo* target data for FinO crucially complement available data sets for the *Salmonella* FopA and ProQ RBPs (4,6). From this, a picture is emerging wherein three FinO domain RBPs operate in the *Salmonella* cytosol to serve regulatory functions both in the chromosome and on different extrachromosomal elements. This also includes the novel observation that an RBP encoded by one plasmid engages in the regulation of another plasmid.

### Importance of studying bacterial RBPs with global methods

Our study underpins the importance of comprehensive target profiling of bacterial RBPs *in vivo*, as originally pioneered with Hfq (38,61,62). This global approach has turned out to be particularly fruitful for FinO domain proteins. For example, *E. coli* ProQ was originally described as a regulator of ProP, a protein involved in proline uptake (63). While subsequent studies focusing on its FinO domain demonstrated general RNA binding capabilities *in vitro* (64), ProQ continued to be regarded as a protein of specialized function until RIP-seq established it as a truly global RBP in both *E. coli* and *Salmonella* (6,7). When it comes to FopA, the FinO domain protein encoded by *Salmonella* plasmid pCol1B9, it was a RIP-seq experiment that proved this protein to be a functional RBP. Intriguingly, although FopA was close to FinO in terms of amino acid sequence, it showed a distinct suite of targets dominated by Inc RNA, which is a pCol1B9-encoded 70-nt antisense transcript with little if any similarity to FinP or RepX (4).

In *Legionella*, while a role of RocC in natural transformation had been predicted through a genetic screen, RIP-seq uncovered the molecular function of RocC as a post-transcriptional mRNA repressor of competence genes (10). Lastly, UV CLIP-seq proved a straightforward approach to answering the fundamental question whether the short *Neisseria* ProQ protein, which consists solely of a FinO domain, would act as a specialized or a global RBP (9). Therefore, given the large diversity and poor predictability of the *in vivo* targets of FinO domain proteins, it seems highly advisable to begin studies of new members of this RBP class by a quick profiling of its *in vivo* ligand using RIP-seq or a related method such as UV CLIP-seq or RIL-seq (7,8). Ideally, such early target profiling should include different stages of growth or diverse growth conditions, as illustrated by the impressively different profiles of ProQ ligands along the growth curve (Figure 2D). Even in the case of FinO, RepX might easily be missed when profiling the exponential phase alone (Figure 2D), especially when knowledge or the annotation of the plasmids carried by a bacterium of interest is incomplete. Homologs of FinO/ProQ are found in a plethora of other species such *Pseudomonas*, *Vibrio* or *Klebsiella* (2,3,65). Uncovering its RNA targetome will shed additional light on protein features determining RNA target specificity.

As to comparing the RIP-seq results of FinO and ProQ (Figures 1 and 2), the specificity of FinO for its targets of RepX and FinP does not seem to arise from having fewer moderate interactors, as compared to ProQ. This may suggests that FinO achieves RNA specificity relative to ProQ, not by binding to fewer RNAs moderately well but by binding to two RNAs much better than ProQ binds any of its targets. However, a comparison of the binding strengths of these RBPs as they interact with cellular RNAs is not trivial. To address this question, it will be important to develop genuine *in vivo* binding assays determining inside cells whether, for example, FinO binds RepX more strongly than ProQ binds the SibA RNA. Factors such relative concentrations of the very target RNA (and other target RNAs of the same protein present in the cytosol) and the protein of interest will have to be taken into account as well.

### *Salmonella* as a model bacterium with three FinO domain proteins

**Figure 7.**
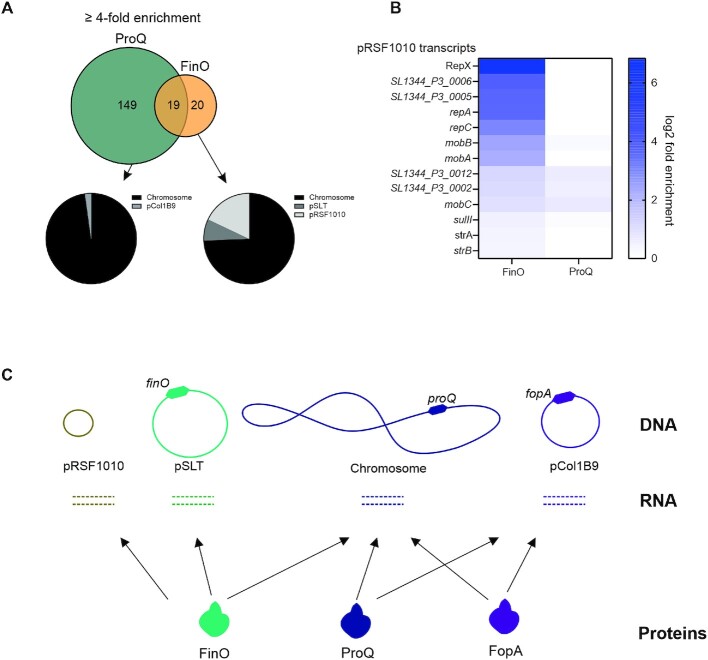
FinO and ProQ targetome in *Salmonella enterica* SL1344. (**A**) Venn diagram depicting overlap of transcripts enriched over 4-fold by FinO and ProQ. Genetic loci localization of transcripts bound by FinO and ProQ. (**B**) Heat map indicating enrichment of transcripts encoded in pRSF1010 by FinO and ProQ. FinO enriches for 7 out of 13 loci encoded in pRSF1010. (**C**) Model diagram indicating genetic location of FinO, ProQ and FopA in *Salmonella enterica* SL1344 genetic elements.

The *Salmonella enterica* strain SL1344 used here, which carries three different plasmids and expresses three distinct FinO domain proteins (Figure 1A) whose *in vivo* RNA ligands are now well-established, should be an excellent model to study the target selectivity of these RBPs. Of the three RBPs in question, FinO primarily interacts with transcripts from plasmids pSLT and pRSF1010, as well as from the chromosome, but not with transcripts of plasmid pCol1B9 on which FopA is encoded (Figure 7A-B). By contrast, FopA has strong selectivity for transcripts from its own plasmid and additionally associates with mRNAs and sRNAs from the *Salmonella* chromosome; whereas ProQ shows the reciprocal behavior, with the vast majority of its *in vivo* ligands coming from the chromosome while also binding transcripts from pCol1B9 (Figure 7C). Naturally, we caution that these patterns are not fully quantitative, and whether an RNA association translates into physiological relevance is another question. However, studies of Hfq-associated sRNAs generally support a view that enrichment of a cellular transcript in a RIP-seq experiment usually indicates a functional relationship (target or sponge) with the RBP in question (38). In the same vein, our observation of an altered copy number of pRSF1010 in the Δ*finO* strain indicates that at least some of these observed RNA-protein associations between chromosome and plasmids could have a regulatory function.

A good *in vivo* model is important in light of the fact that *in vitro* some of these proteins can show micromolar affinities to their RNA targets (4,13,27), which can hardly be reconciled with their observed selectivity and successful discrimination of abundant rRNA and tRNA. For example, FinO and ProQ show similar *in vitro* affinities for the FinP sRNA (64), whereas *in vivo* FinP is selectively bound by FinO (Figure 2), although the intracellular concentration of ProQ far exceeds that of FinO, and although the FinP sRNA seems more abundant than other individual ligands of ProQ.

What is the reason for this strong discrepancy between *in vivo* and *in vitro* interactions? Possible partner proteins, which might influence RNA binding *in vivo*, are unknown for both FinO and ProQ. In addition, even though ProQ possesses a C-terminal extension as compared to FinO, structural and genetic studies suggest that RNA binding is chiefly determined by the N-terminal FinO domain (12,13,29). Fortunately, *Salmonella enterica* and its plasmids are genetically tractable, thus this bacterium lends itself to domain and gene swapping experiments. Will the FinO domain of FinO, when replaced with that of ProQ, still support plasmid conjugation and replication by preferably binding FinP and RepX as well as their antisense targets? FinO strongly enriches FinP and RepX while ProQ has a broader targetome (Figure 1C). The limited targetome of FinO might be due to an intrinsic FinO limitation or alternatively, the presence of high affinity targets, not present for ProQ, might precludes FinO to have a more broad function. Will FinO interact with other transcripts when FinP and RepX are genetically removed?

Intracellular localization might also play a role, as there is increasing evidence for spatial control of gene expression in bacteria (66,67). For example, will ProQ expressed from plasmid pSLT show the same target profile as when made from its native chromosomal gene? Vice versa, will FinO when expressed from the chromosome also primarily associate with the plasmid-encoded FinP and RepX sRNAs? Whether all three proteins are equally dispersed in the bacterial cytosol or show distinct localization is currently unknown. What we do know is that the pSLT plasmid localizes non-randomly in the cell (68), that pSLT plasmid copy number varies dramatically in individual cells (68), and that thanks to its control by Dam methylase the synthesis of the FinP sRNA will also be variable on the single-bacterium level (69,70). Obviously, all these factors which might affect the rate of FinO-RNA association and its functional consequences.

### How the FinP–RepX pair might help to crack the RNA recognition code of the FinO domain

One of the most intriguing aspects of the target suites of FinO domain RBPs is the continued absence of an obvious sequence consensus amongst the bound RNAs. Nonetheless, there is a clear trend for these target RNAs to be structured, as previously calculated by comparing the sRNAs bound by Hfq and ProQ (6). It thus appears that the FinO domain recognize some abundant type of RNA structure or shape. Unfortunately, despite considerable effort (29,30,71,72), a high-resolution protein structure with a natural RNA target is not yet available for any member of this RBP class. Therefore, bottom-up mutational analysis of established RNA ligands remains an important strategy for determining which RNA features are recognized by the FinO domain (7,13). We believe that the new FinP/RepX sRNA constitutes an outstanding model in this regard. FinP and RepX are largely identical in length and structure, but share only 50% of nucleotide sequence, and neither of them resembles any of the top-enriched transcripts of ProQ (Supplementary Figure S5). Recently developed high-throughput methods such as RNA Bind-n-Seq (73) should provide a cost-effective approach to resolving the sequence and secondary structure preferences of FinO. In such an experiment, one would challenge a preformed FinO–RepX complex with a pool of *in vitro*-synthesized RNAs with mutations of all bases that differ from FinP, essentially seeking to ‘morph’ RepX into FinP. We would expect RNA variants that succeed at outcompeting RepX to become enriched on FinO, which could then be read out by deep sequencing. Such an approach may yield high-affinity RNA variants that are neither FinP nor RepX, to increase the starting pool for FinO-RNA co-crystallization efforts in a rational manner.

Despite their overall similarity, there are notable differences between RepX and FinP. RepX is lacking the 5′ extension and its short 3′ tail does not show the three consecutive uridines of FinP (Figure 4B). Importantly, both the 5′ extension and the 3′ uridine tail are known to promote FinO recognition of FinP (27). Moreover, a recent *in vitro* analysis of mutant RNAs of the *E. coli malM* 3′-UTR and the FinO domain of ProQ N-terminal domain concluded that for tight RNA binding, a terminator hairpin of at least 2 bp followed by a 3′ oligouridine tail is required (13). In a preliminary experiment, we mutated the 3′ tail of RepX to a three-uridine end of FinP, yet did not observe a better binding to FinO (Supplementary Figure S6). Thus, the case of RepX suggests that other yet-unrecognized RNA features, perhaps the overall shape, strongly contribute to binding, at least in the very case of FinO. A systematic analysis of hybrid molecules of FinP and RepX will be required to understand why FinO favours FinP over the similar RepX sRNA.

### Cross-regulation of plasmid replication

Plasmids are generally known as selfish genetic elements that spread independently, with the exception of large virulence plasmids whose transfer between bacteria by conjugation can depend on specialized proteins from another (helper) plasmid in the same cell. Our present work provides preliminary evidence for a new type of plasmid interdependency, where one plasmid influences the replication rate of another, as suggested by an altered the copy of plasmid pRSF1010 after inactivation of the *finO* gene in plasmid pSLT. For background, the RepX sRNA was originally identified as a 75-nt RNA antisense to the ribosome binding site of *repA-repC* mRNA in pR1162 plasmid, which is parental to pRSF1010 (58). Mutations in the RepX sRNA cause an increase in plasmid copy number, suggesting that RepX acts to repress the synthesis of the replication protein RepA (58) or alternatively by downstream effect on the RepC replication protein expression (59,60). In our study, we observe an increase in the copy number of pRSF1010 in a Δ*finO* strain, which we propose to result from the reduced steady-state levels of RepX in this background.

Interestingly, the RepX transcript is strongly conserved among members of the IncQ-1 family of plasmids to which pR1162 and pRSF1010 belong. This raises the intriguing possibility that FinO of pSLT, which belongs to the IncF family of plasmids, would be able to interact with the RepX sRNA of all IncQ-1 plasmids. Of note, IncQ-1 plasmids while not self-transmissible are generally mobilizable thanks to their ability to hijack the type IV conjugation machinery of self-transmissible plasmids (57). Therefore, one could hypothesize that FinO in addition to help controlling the TraJ conjugation factor of plasmid pSLT, acts on the replication locus of a cohabitating plasmid that wants to utilize the pSLT conjugation machinery for its own mobilization. Interestingly, plasmid pRSF1010 is mobilizable to a high frequency by IncP or IncX plasmids. However, it does not compete well for the type IV secretion system encoded by the IncF plasmids (57,74), one reason for that being that one of the conjugation machinery's proteins moonlights to restrict the access of pRSF1010 (75). By binding to RepX and other transcripts of pRSF1010 (Figure 7B), the FinO proteins of IncF plasmids might act in parallel to further suppress cohabitating IncQ-1 plasmid on the level of replication. Alternatively, the repressor function of FinO might become important after successful conjugation of both plasmids, keeping the copy number of the second plasmid down in order to reduce the metabolic burden imposed on the recipient cell to insure maintenance of the pSLT plasmid (76). While the possibility remains that the effect of FinO on pRSF1010 is fortuitous or even RepX-independent, our observation that one plasmid influences the copy number of another through its main RBP clearly warrants a more detailed analysis, also against the backdrop that new molecular strategies for preventing the spread of antibiotic resistance plasmids are needed.

## DATA AVAILABILITY

The sequencing data have been deposited in NCBI’s Gene Expression Omnibus (77) and are accessible through GEO Series accession number GSE166112.

## Supplementary Material

gkab281_Supplemental_Files
