# Supplementary material for: In vivo targets of Salmonella FinO include a FinP-like small RNA controlling copy number of a cohabitating plasmid
Source: Nucleic Acids Res. 2021 May 3;49(9):5319–35. doi: 10.1093/nar/gkab281 (PMC8136791; doi:10.1093/nar/gkab281)

**Supporting material for**

"*In vivo targets of Salmonella FinO include a FinP-like small RNA controlling copy number of a cohabitating plasmid*"

Youssef El Mouali, Milan Gerovac, Raminta Mineikaite, Jörg Vogel

## Supplementary figures legends

**Figure S1.** RIP-seq of FinO-3×FLAG and ProQ-3×FLAG. **A.** Western blot of *Salmonella enterica* containing FinO-3×FLAG. **B.** Read distribution of WT, FinO-3×FLAG and ProQ-3×FLAG RIP-seq samples shown in Fig. 1C. **C.** PCA plot distribution of duplicate samples of WT, FinO-3×FLAG and ProQ-3×FLAG RIP-seq samples shown in Fig. 1C.

**Figure S2.** Alignment of FinP and RepX sequences. **A.** Alignment of RepX sequences detected by GLASSgo search with RepX from pRSF1010_SL1344 as input. The NCBI GenBank accession number coordinates and length of the sequences is indicated. **B.** Alignment of FinP sequences detected by GLASSgo search with FinP from pSLT_SL1344 as input. The NCBI GenBank accession number, coordinates and length of the sequences is indicated. Sequences were aligned by LocARNA and visualized in Jalview.

**Figure S3.** Secondary structures of FinP and RepX sRNAs and *traJ* and *repA* mRNA's 5'UTR. RNA sequences of FinP, RepX and their antisense sequences on *traJ* and *repA* mRNA were folded in RNAfold. The resulting structures were visualized by Varna. Residues of the Shine-Dalgarno (SD) and Anti-SD regions are highlighted in blue. Start codons and anti-start codon are highlighted in yellow.

**Figure S4. FinP and RepX competition for FinO binding. A.** EMSA with FinP competing FinP* from FinO binding. Radiolabeled FinP (4 nM) was incubated with increasing concentration of FinO (0, 63, 125, 250, 500 nM). In the presence of 500 nM of FinO, the binding was competed with increasing concentration of non-radiolabeled FinP (0.13, 0.25, 0.5, 1, 2 μM). **B.** EMSA with RepX competing RepX* from FinO binding. Radiolabeled RepX (4 nM) was incubated with increasing concentration of FinO (0, 63, 125, 250, 500 nM). In the presence of 500 nM of FinO, the binding was competed with increasing concentration of non-radiolabeled RepX (0.13, 0.25, 0.5, 1, 2 μM). **C.** EMSA with RepX competing FinP from FinO binding. Radiolabeled FinP (4 nM) was incubated with increasing concentration of FinO (0, 0.63, 1.25, 2.5, 5 μM). In the presence of 5 μM of FinO, the binding was competed with increasing concentration of non-radiolabeled RepX (0.13, 0.25, 0.5, 1, 2 μM). **D.** EMSA with FinP competing RepX from FinO binding. Radiolabeled RepX (4 nM) was incubated with increasing concentration of FinO (0, 0.63, 1.25, 2.5, 5 μM). In the presence of 5 μM of FinO, the binding was competed with increasing concentration of non-radiolabeled FinP (0.13, 0.25, 0.5, 1, 2 μM).

**Figure S5. Comparison of ProQ and FinO bound sRNAs structures. A.** Secondary structures of FinO-bound sRNAs of FinP and RepX. **B.** Secondary structures of ProQ-bound sRNAs SibA, Stnc540, SibD, RaiZ, SraL and RyfD. The secondary structures were predicted with RNAfold and visualized by Varna. The targets of FinO, FinP and RepX share a two stem-loop structure. The top targets of ProQ: SibA, Stnc540, SibD, RaiZ, SraL and RyfD are highly structured but more heterogeneous.

**Figure S6. Filter binding assay of RepX and RepX C-U variant. A**. Radiolabeled FinP (4 nM) was incubated with increasing concentration of FinO (0, 0.63, 1.25, 2.5, 5 μM). In the presence of 5 μM of FinO, the binding was competed with increasing concentration of non-radiolabeled RepX (0, 0.13, 0.25, 0.5, 1, 2 μM). **B.** As in **A.** the binding of FinP to FinO was competed with increasing concentration of non-radiolabeled RepX C-U variant that carries a 3'U stretch (0, 0.13, 0.25, 0.5, 1, 2 μM). All RNA transcripts used were obtained by T7 *in vitro* transcription. Samples were filtered in a Dot blot apparatus. FinO bound to

RNA is visualized in a nitrocellulose membrane (top panel) while unbound RNA is visualized in a Hybond+ membrane (bottom panel).

**Supplementary Table S1** Bacterial strains, plasmids and oligonucleotides

**Supplementary Table S2** FinO RIP-seq

**Supplementary Table S3** ProQ RIP-seq

**Supplementary Table S4.** ProQ and FinO RIP-seq growth curve.

# El Mouali et al. Figure S1

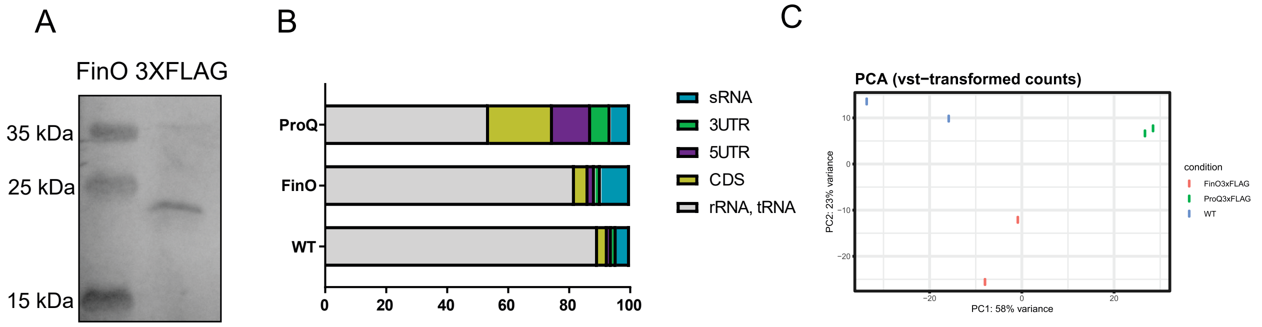

El Mouali et al. Figure S2

A

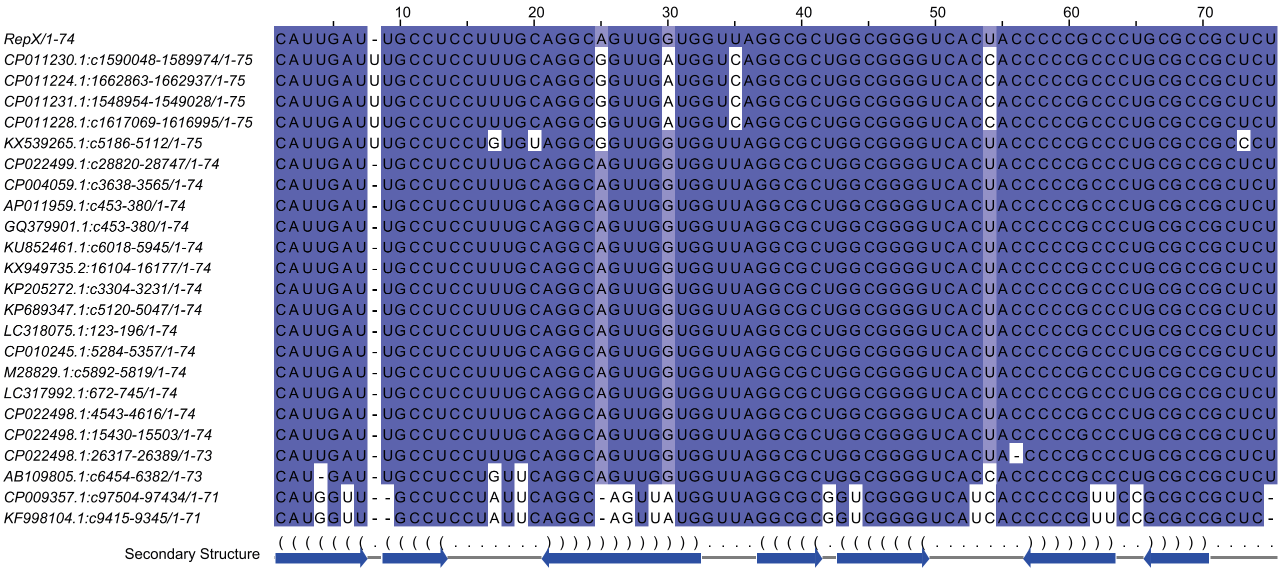


B

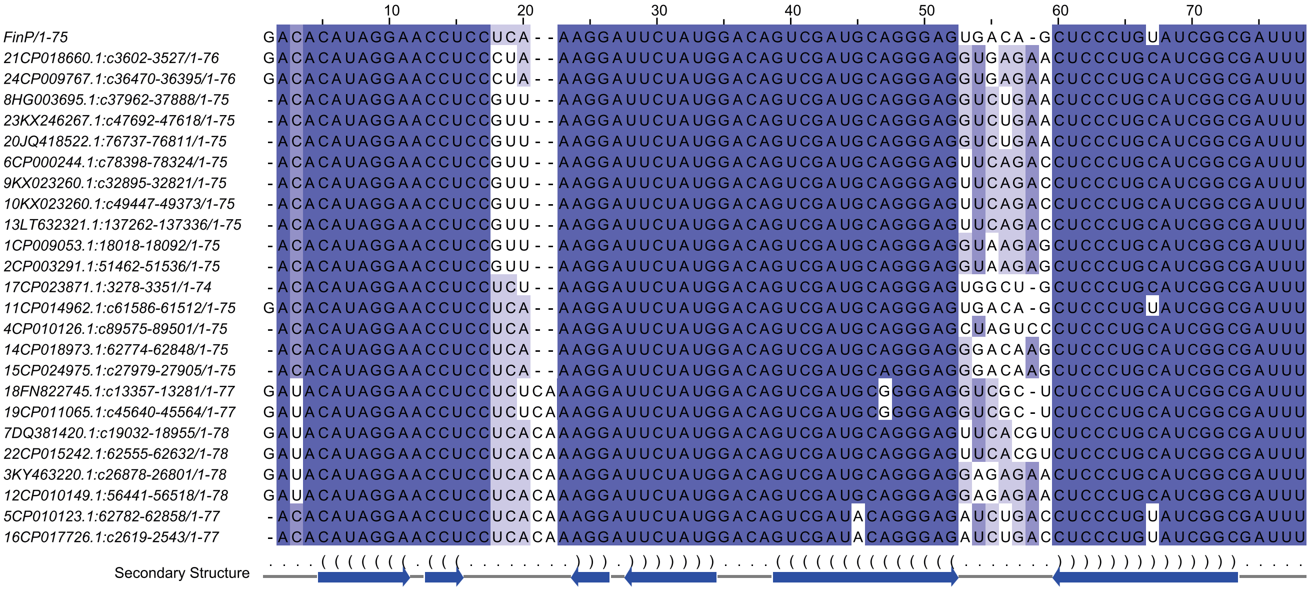

El Mouali et al. Figure S3

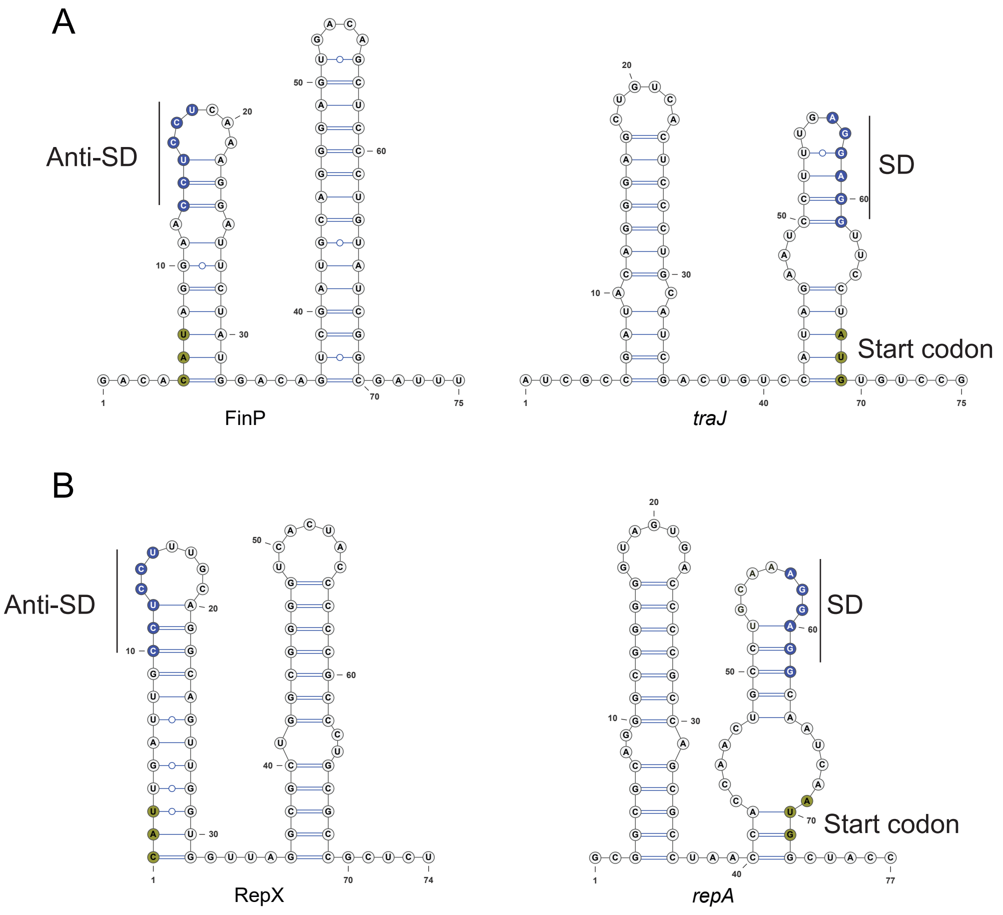

El Mouali et al. Figure S4

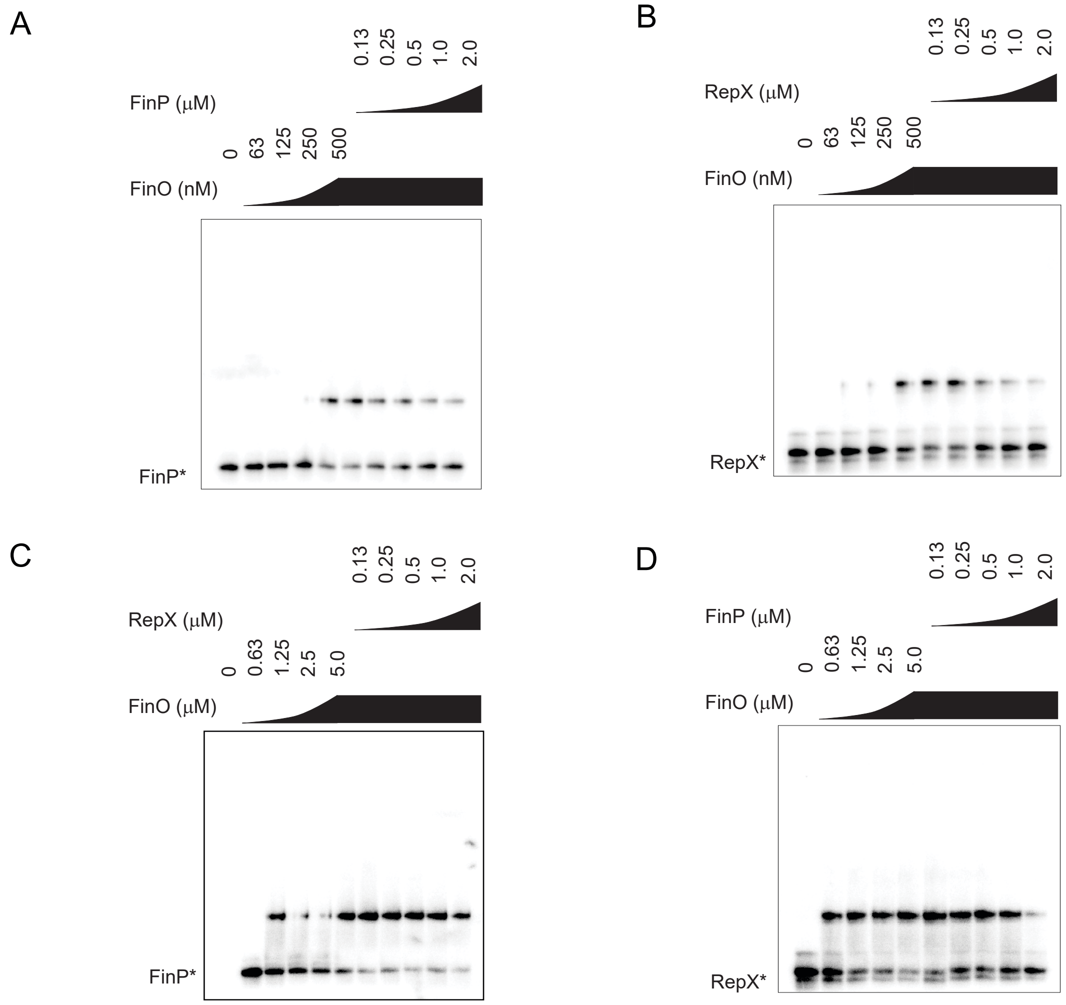

El Mouali et al. Figure S5

A

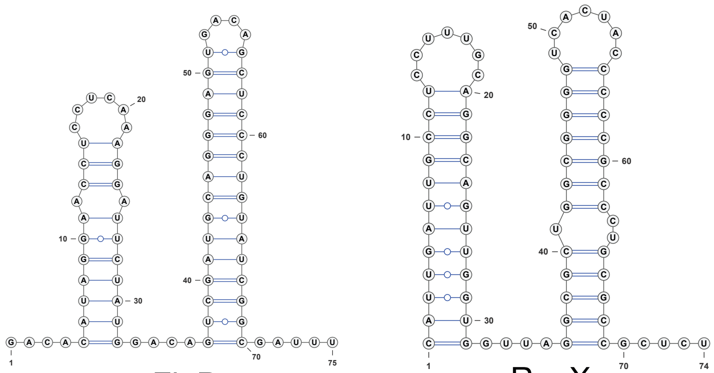

FinP RepX

B

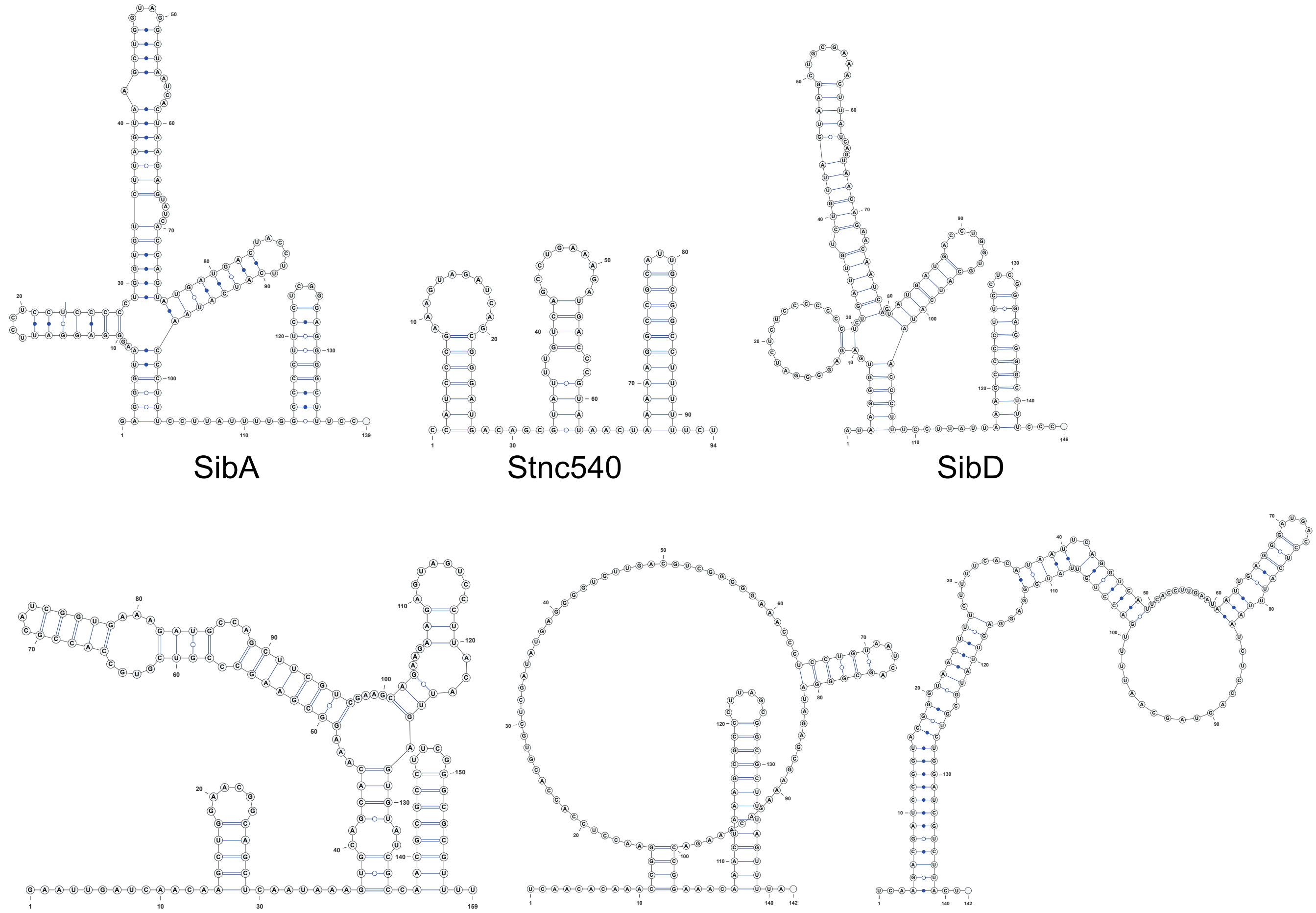

SibA Stnc540 SibD

RaiZ SraL RyfD

El Mouali et al. Figure S6

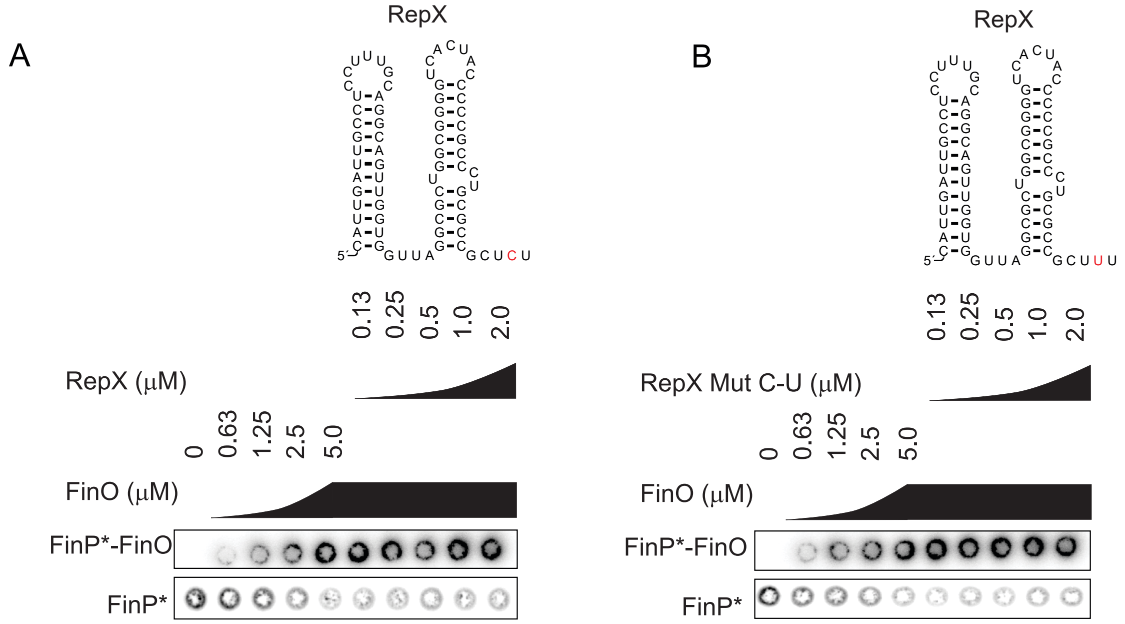

Supplement: gkab281_Supplemental_Files [file gkab281_supplemental_files.zip › Supporting.material.pdf]
